# Influence of Temperature and Sulfate Concentration on the Sulfate/Sulfite Reduction Prokaryotic Communities in the Tibetan Hot Springs

**DOI:** 10.3390/microorganisms9030583

**Published:** 2021-03-12

**Authors:** Li Ma, Weiyu She, Geng Wu, Jian Yang, Dorji Phurbu, Hongchen Jiang

**Affiliations:** 1 State Key Laboratory of Biogeology and Environmental Geology, China University of Geosciences, Wuhan 430074, China; mlll@cug.edu.cn (L.M.); weiyushe@cug.edu.cn (W.S.); yangjiancug@126.com (J.Y.); 2 Tibet Plateau Institute of Biology, Lhasa 850000, China; puduo@126.com

**Keywords:** sulfate/sulfite-reducing prokaryotes, *dsrB* gene, hot spring, temperature, Tibet

## Abstract

The distribution and diversity of sulfate/sulfite reduction prokaryotic (SRP) communities in hot springs from the Quzhuomu and Daggyai Geothermal Zone of Tibetan, China, was reported for the first time. In hot springs that are naturally hyperthermal and anoxic, the sulfur cycle is one of the most active cycles of the elements. The distribution of SRP in response to temperature is of great importance to the understanding of biogeochemical cycling of sulfur in geothermal features. Little is known about the SRP in geothermal zone. In this study, the diversity of SRP was investigated in the sediments from the Daggyai and Quzhuomu geothermal zone using PCR amplification, cloning and sequencing of the dissimilatory sulfite reductase beta subunit gene (*dsrB*). The abundance of *dsrB and* 16S rRNA genes, were determined by quantitative polymerase chain reactions. In addition, correlations of the SRP assemblages with environmental factors were analyzed by the aggregated boosted tree (ABT) statistical analysis. The results showed that SRP populations were diverse, but were mainly composed of *Desulfobacterales*, *Desulfovibrionales*, *Syntrophobacterales*, *Clostridia* and *Nitrospirales*, and large fraction (25%) of novel sequences have branched groups in the *dsrB* phylogenetic tree. In Quzhuomu geothermal zone, sulfate-rich hot springs are characterized by thick bacterial mats that are green or red and the SRP populations mainly appear at mid-temperature (50 °C to 70 °C). In low-sulfate hot springs in the Daggyai geothermal zone, although gray or pink streamers are widely formed at 60 °C to 80 °C, they prefer to inhabit in green mat at lower temperature (30 °C to 50 °C). With increasing temperature, the diversity of the *dsrB* gene at the OTU level (cutoff 97%) decreased, while its relative abundance increased. This result suggests that temperature played an important role in affecting *dsrB* gene distribution.

## 1. Introduction

Sulfate respiration is one of the oldest ways for microorganisms to acquire energy on the Earth [1,2]. As part of the global S cycle, biological sulfate reduction is ubiquitous in the Earth’s anaerobic environments and is essential basis of the biosphere [3,4]. For example, a majority (up to 97%) of the sulfide produced on the Earth is attributable to the activity of sulfate-reducing prokaryotes (SRPs) in mesophilic environments [5,6,7,8,9]. In addition, SRP gain energy for cell synthesis and growth by coupling the reduction of sulfate (SO_4_^2−^) to sulfide (H_2_S, HS^−^) with cycling of carbon and nitrogen [10,11,12], and play an important role in arsenic geochemistry transformation [13]. Studying the diversity of SRP communities and the environmental variables affecting it is of great importance for understanding the biogeochemical cycle of sulfur.

Most known SRPs contain sulfate reductase, which catalyzes the transformation of sulfate to sulfide [14,15]. The large subunit of sulfate reductase is encoded by the conservative *dsrAB* gene, a widely accepted gene marker for studying SRP distribution and activity in various environments, such as freshwater lake sediment [16], hot springs [17], marine sediments [18,19,20,21,22], hypersaline soda lakes [23], paddy soils [24], acid mine drainages [25], deep-sea hydrothermal vents [26], freshwater wetlands [27], aquifer environments [28], estuarine sediments [29], and deep terrestrial subsurface [30]. These previous studies showed that SRP were diverse in various environments and their sulfate reduction activity could be correlated with environmental factors such as temperature, pH and water chemistry [18,22,31]. For example, temperature affects diversity and sulfate reduction and/or growth rates of SRP in sediments, and SRP cultures exhibit typical asymmetric curves and may follow the Arrhenius function over a certain range of temperature below optimum [8,32,33,34,35,36]. Most of the above-mentioned SRP studies were performed under ambient conditions in the 0–40 °C temperature range. However, little is known about the abundance and diversity of sulfate-reducing bacteria (SRB) and their response to environmental factors (e.g., temperature) in geothermal features (>45 °C). Thus, a holistic survey of SRP diversity and its response to environmental factors (e.g., temperature) is essential.

As the largest and highest plateau on the Earth, the Tibetan Plateau is well known for its volcanic activity and geothermal features, among which the Daggyai and Quzhuomu geothermal zones host a number of hot springs at a high elevation (>4500 m above sea level). These hot springs are characterized by a wide range of temperature (up to 97 °C). Compared to other studies on terrestrial hot springs at lower elevations, so far available microbiological data is limited in the Tibetan hot springs [37,38,39,40]. Previous studies showed that microbes were abundant and diverse in the Tibetan hot springs and temperature played an important role in affecting the distribution of bacterial, archaeal and arsenite-oxidizing bacterial communities in the Tibetan hot springs [41,42,43]. However, to date, little is known about the distribution of the SRP in these springs and how SRP populations vary with environmental factors.

The major objective of the present study was to investigate the abundance and diversity of SRP and their correlation with environmental factors in the Daggyai and Quzhuomu geothermal zones of the Tibetan Plateau. An integrated approach was employed including geochemistry, quantitative polymerase chain reaction (qPCR) and *dsrB* gene-based phylogenetic analyses.

## 2. Methods and Materials

### 2.1. Field Measurements and Sample Collection

**Figure 1:**
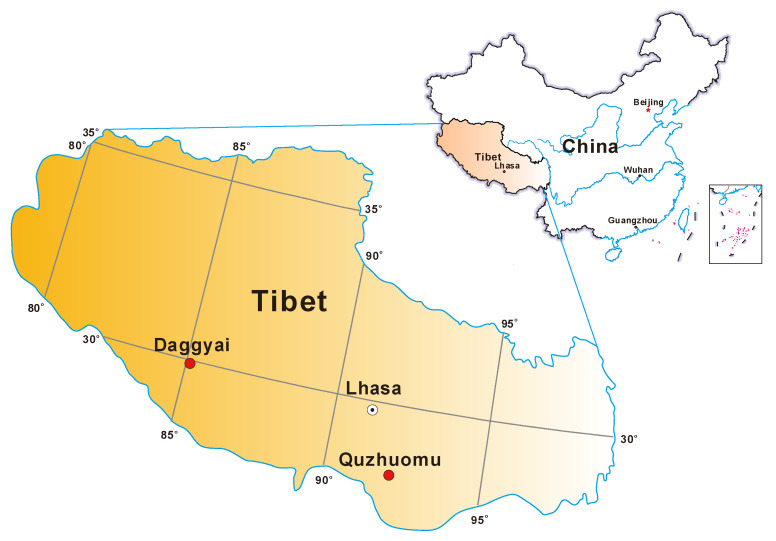
The locations of the Daggyai and Quzhuomu Geothermal Zone in the Tibetan Plateau.

In August 2015, field measurements and sample collections were performed in the hot springs in Daggyai (DG) geothermal zone in Shigatse city and Quzhuomu (QZM) geothermal zones in Shannan City, respectively (Figure 1). A total of 15 samples, including seven from individual hot springs and eight from the outflowing channels of the sampled hot springs, were selected for this study (Table 1). In the Daggyai geothermal zone, the five sampled individual hot springs were labeled with DG-1, -4, -5, -14 and -16; In the Quzhuomu geothermal zone, the collected samples along the outflowing channels of two hot springs were labeled with QZM-4, -5, -6, -7 and QZM-9, -10, -11, -12, respectively; the other two individual hot springs were labeled as QZM-13 and QZM-14. At each sampling site, water temperature, pH, dissolved oxygen (DO) and Fe^2+^ were measured in the field with a temperature probe (LaMotte, Chestertown, MD, USA) and Hach meter (equipped with pH, Fe^2+^ and dissolved oxygen sensors, Hach Company, Loveland, CO, USA), respectively. For dissolved organic carbon (DOC) analysis, hot spring water was filtered through pre-combusted (450 °C, 4 h) GF/F filters (0.7 mm pore size, Whatman, Buckinghamshire, UK), and the resulting filtrate was collected into pre-combusted brown glass bottles with addition of concentrated phosphoric acid (final conc. 0.2% (*v*/*v*)), 25 mL hot spring water was filtered through 0.22 µm nitrocellulose membranes and collected into acid-washed polyethylene bottles for major anion especially sulfate concentration measurement, 25 mL hot spring water was filtered through polycarbonate membrane filters (pore size 0.22 mm) and collected into glass bottles supplemented with concentrated HNO_3_ (to a final concentration of 0.1 M) for major cation measurement measurement. Sediments for geochemical and microbial analyses were collected in 50-mL Falcon tubes, and were then frozen in dry ice in the field and during transportation. Once in the laboratory, the sediment samples were transferred in a −80 °C freezer until further analysis.

### 2.2. Geochemistry Analyses

Major cation and anion concentration of the hot spring water was measured using ion chromatograph (IonPac AS18 4 × 250 mm for anion, ICS 600, ThermoFisher, Carlsbad, CA, USA). The DOC and TOC contents were measured by using an NC 2100 Elemental Analyzer (multi N/C 2100, Analytic Jena, Jena, Überlingen, Germany). Before TOC measurements, the sampled sediments were fumigated with HCl to remove carbonates.

### 2.3. DNA Isolation, PCR Amplification, and Phylogenetic Analyses

DNA was extracted from the collected sediment samples by using a FastDNA Spin Kit for Soil (MP Biomedicals, LLC, Solon, OH, USA) according to the manufacturer’s instructions. The sulfate-reducing prokaryotic *dsrB* genes were amplified from the extracted DNA samples with the primer set of DSR-p2060F (5′-CAA CAT CGT YCA YAC CCA GGG-3′) and DSR-4R (5′-GTG TAG CAG TTA CCG CA-3′) according to the conditions as reported previously [44]. The polymerase chain reaction (PCR) products were purified with the AxyPrep DNA Gel Extraction Kit (Axygen Scientific Inc., Union City, CA, USA), followed by ligation into pGEM^®^-TEasy Vector (Promega, San Luis Obispo, CA, USA) and, a total of 15 *dsrB* gene clone libraries (one for each sample) were constructed. From each clone library, 20–40 clones were randomly selected for sequencing with the primer M13. The obtained raw nucleotide sequences were checked and trimmed manually. Potential chimeric sequences were removed from further analysis. The operational taxonomic units (OTUs) of the *dsrB* gene clone sequences were determined based on a cutoff value of 98% by using DOTUR [45]. Coverage (C) of the clone libraries was calculated as follows: C = 1 − (*n*1/*N*), where *n*1 is the number of OTUs that occurred only once in the clone library and *N* is the total number of clones analyzed [46]. One representative sequence from each OTU was selected for downstream phylogenetic analysis and then was translated into amino acid sequences. The resulting amino acid sequences were Blasted against available *dsrB* gene amino acid sequences in the GenBank database (http://www.ncbi.nlm.nih.gov (accessed on 5 March 2021)). Closest references were chosen for further phylogenetic analysis. A neighbor-joining phylogenetic tree of inferred *dsrB* amino acid sequences was constructed with the Poisson model in the MEGA 5.0 software [47]. Diversity indices (Shannon and Simpson) were calculated by the PAST software package version 2.09 [48]. Correlations of the SRB assemblages with environmental factors were analyzed by the aggregated boosted tree (ABT) statistical analysis. In order to accurately predict and explain the relationships between ecological data and environmental variables, aggregated boosted tree (ABT) analysis was performed by using R package “gbm” [49].

### 2.4. Quantitative Polymerase Chain Reaction (qPCR)

The abundance of *dsrB* and bacterial 16S rRNA genes was determined by using qPCR with primer sets of DSR-p2060F/ DSR-4R and BACT1369F (5′-CGG TGA ATA CGT TCY CGG-3′)/PROYK1492R (5′-GGW TAC CTT GTT ACG ACT T-3′), respectively. Amplification conditions for *dsrB* gene were 94 °C for 15 min, followed by 40 cycles (30 s at 94 °C, 30 s for annealing at 58 °C, and 30 s at 72 °C), and the amplification conditions for 16S rRNA gene were 95 °C for 15 min, followed by 40 cycles (15 s at 95 °C, 30 s for annealing at 58 °C, and 20 s at 72 °C). Each reaction volume was 25 μL, containing 12.5 μL of QuantiTect SYBR-Green Master Mix (QIAGEN, Valencia, CA, USA) and 2.5 pmol of each primer. Purified plasmid DNA of *dsrB* and 16S rRNA genes was used for construction of standard curves. For template preparation of standard curves, purified plasmid DNA was 10-fold serially diluted ranging from 1.8 × 10^2^ to 1.8 × 10^8^ gene copies per microliter. All qPCR reactions were performed in triplicate. qPCRs were performed on an ABI7500 real-time PCR system (Applied Biosystems, Carlsbad, CA, USA). R^2^ value of the qPCR standard curves was 0.999, and the qPCR reaction efficiency was 96–102%. The quality and length of the qPCR products were checked by dissociation curve analysis and 1% agarose gel electrophoresis, assuming that one bacterial cell contained 3.6 copies of 16S rRNA gene and one SRB cell contained one copy of *dsrAB* gene [50,51]. The qPCR results were expressed as gene copies per gram (copies g^−1^) sediments.

## 3. Results

### 3.1. Geochemical Characteristics of the Studied Hot Springs

In the present study, the temperature of the sampled hot springs ranged from 32–82 °C, and pH was nearly neutral (6.2–8.0) (Table 1). In general, the sampled hot springs from the Daggyai geothermal zone had lower concentrations of SO_4_^2−^ (8.43–83.43 mg∙L^−1^ vs. 252.7–492 mg∙L^−1^) and Fe^2+^ (0.01–0.2 vs. 0.05–2.39 mg∙L^−1^) than those from the Quzhuomu geothermal zone, and the major cation and anion concentration of the hot spring water were showed in Table S1.

### 3.2. dsrB Gene Diversity in the Studied Hot Springs

**Figure 2:**
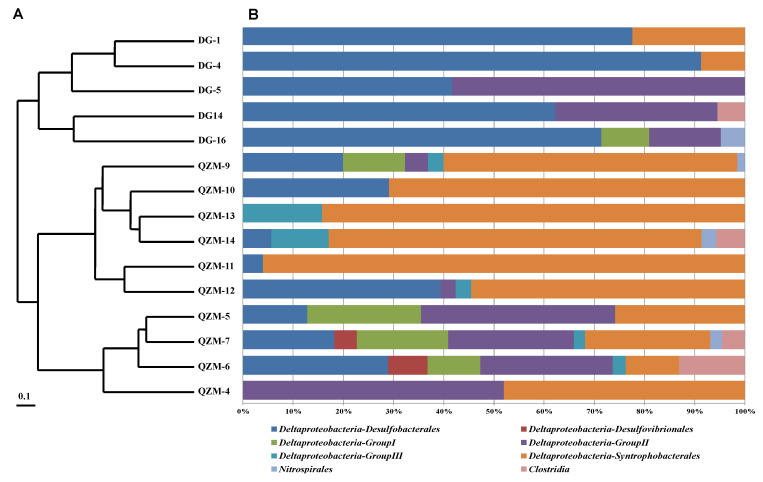
Cluster analysis of the *drsB* gene population composition in the investigated hot spring sediments based on Bray–Curtis dissimilarity. The topology was constructed with the pair group algorithm using the PAST software package (**A**). (**B**) represents community structures, showing the frequencies of clones affiliated with major phyla.

A total of 497 *dsrB* gene clones were retrieved from the 15 samples, and they were classified into 41 OTUs (Table S2). The coverage of the clone libraries ranged from 90.3% to 100% (Table 2). All of the obtained *dsrB* gene clone sequences were affiliated with the Bacteria domain and fell into *Deltaproteobacteria*, *Nitrospirales*, and *Clostridia* with each accounting for 97%, 0.8%, and 2.2% of the total obtained *dsrB* gene clones, respectively (Figure 2); *Deltaproteobacteria* predominated in studied hot springs. The obtained *dsrB* gene OTUs affiliated with *Deltaproteobacteria* could be divided into six clades, i.e., *Desulfobacterales*, *Desulfovibrionales*, *Deltaproteobacteria*-Group I, -Group II, and -Group III, and *Syntrophobacterales*, respectively. The *dsrB* gene OTUs affiliated with *Desulfovibrionales* were only detected in QZM and they were closely related to known sulfate reducers. *dsrB* gene OTUs affiliated with *Syntrophobacterales,* Group I, Group II and Group III were detected in both QZM and DJ. *Syntrophobacterales* was comprised of 38% of the total *dsrB* genes, which were closely related to those retrieved from Three Gorge Reservoir, Evander Mine and Mafic sill (Figure S1) [29]. The *dsrB* gene OTUs affiliated with the unclassified Group I, Group II and Group III showed no relationship to any known *dsrB* gene sequences and thus the unclassified Group I, Group II and Group III may be novel *Deltaproteobacteria* lineages.

### 3.3. 16S rRNA and dsrB Gene Abundance in the Studied Hot Springs

**Figure 3:**
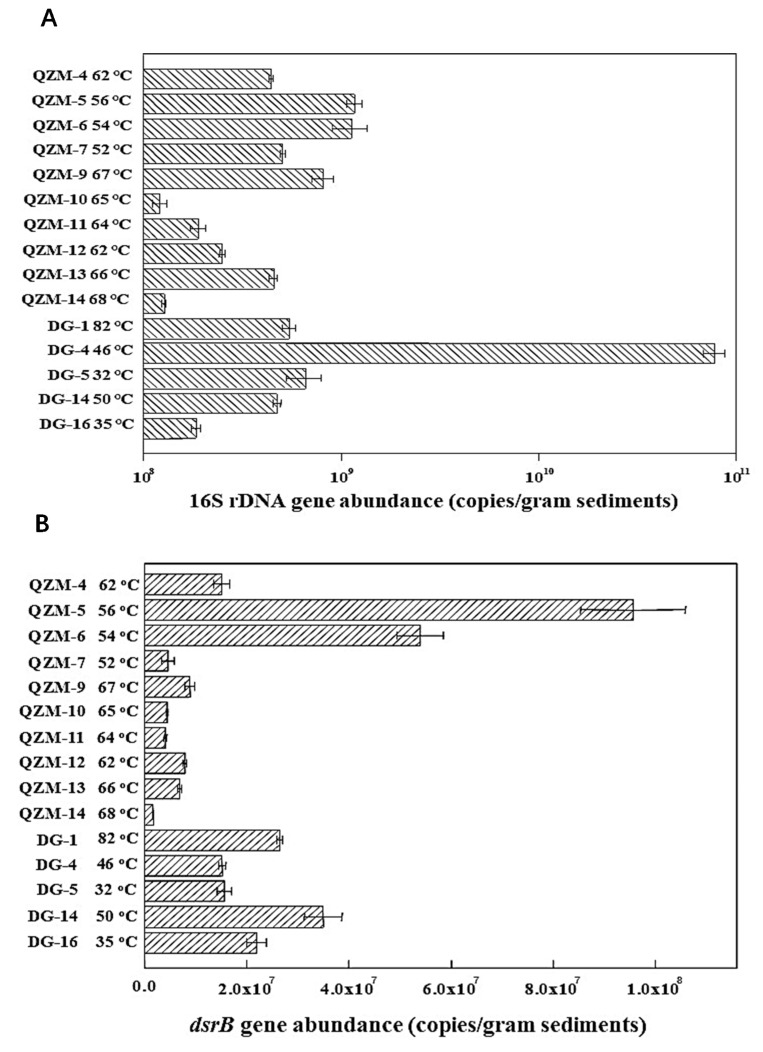
Bacterial 16S rRNA (**A**) and *dsrB* gene (**B**) abundances in the studied hot springs as detected by quantitative polymerase chain reaction (qPCR).

In Daggyai Geothermal Zone, the abundances of 16S rRNA and *dsrB* gene ranged between 1.85 × 10^8^ − 7.72 × 10^10^ and 1.52 × 10^7^ − 3.50 × 10^7^ copies per gram of sediment in the studied hot springs, respectively (Figure 3). In the Qzhuomu Geothermal Zone, the abundances of 16S rRNA and *dsrB* gene ranged 1.21 × 10^8^ − 1.17 × 10^9^ and 1.76 × 10^6^ − 9.56 × 10^7^ copies per gram of sediment in the studied hot springs, respectively (Figure 3). The proportion of SRPs in the bacterial community in Daggyai and Quzhuomu geothermal zone were 0.07–42.6% and 3.3–29.4%, respectively. These data implied that SRB constituted a significant proportion of the bacterial community in the studied Tibetan hot springs.

### 3.4. Impact of Environmental Factors on the Distribution of dsrB Gene in the Studied Hot Springs

**Figure 4:**
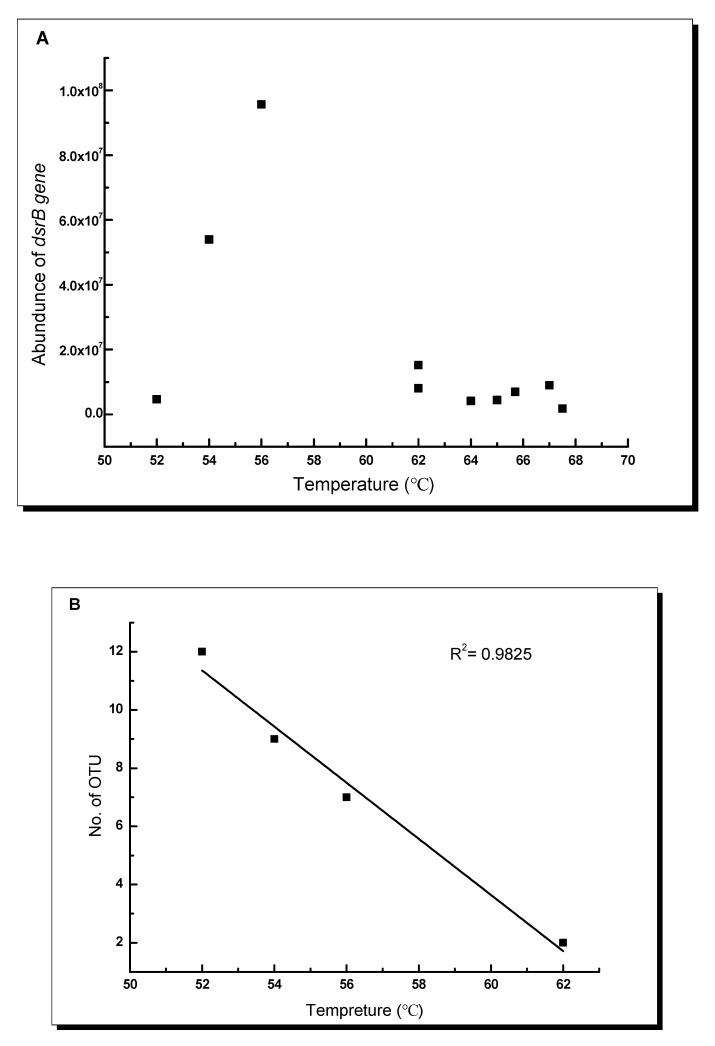
Correlations between temperature and the *dsrB* gene abundance of the studied Tibetan hot springs channel I and II (**A**); correlation between temperature and the number of identified *dsrB* gene operational taxonomic units (OTUs) in the samples along the channel I (i.e., Quzhuomu (QZM)-4, QZM-5, QZM-6, QZM-7) (**B**).

**Figure 5:**
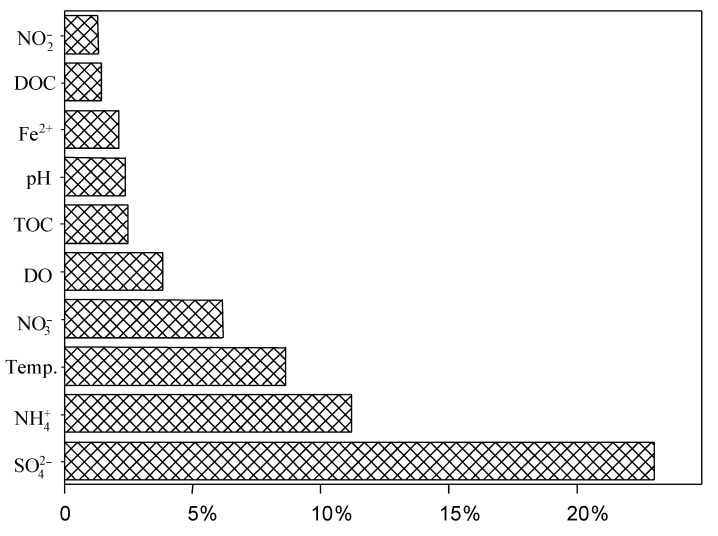
Correlations of the sulfate-reducing bacteria (SRB) assemblages with environmental factors were analyzed by the aggregated boosted tree (ABT) statistical analysis.

Temperature was significantly correlated with the proportion of SRB but not with total bacterial community abundance: in the QZM Geothermal zone, when temperature was below 60 °C, the proportion of SRB showed significantly positive correlation with temperature (R^2^ = 0.9988); when the temperature was higher than 60 °C, the proportion of SRB showed significantly negative correlation with temperature (R^2^ = 0.6248). In channel I (QZM-4, 5, 6, 7, source was QZM 4), the OTU number was negatively correlated with the temperatures (R^2^ = 0.98) (Figure 4). In general, ABT analysis showed that several environmental factors such as temperature and concentrations of SO_4_^2−^ and NH_4_^+^ impacted the distribution of *dsrB* community, the SO_4_^2−^ concentration was the most important impact factor shaping the distribution of *dsrB* gene diversity (Figure 5).

## 4. Discussion

### 4.1. SRP Diversity in the Tibetan Hot Springs

It is notable that none of the retrieved *dsrB* gene sequences in the studied Tibetan hot springs was affiliated with known sulfate reducing archaeal strains or sequences. Previous studies showed that SRP are affiliated with bacteria (*Deltaproteobacteria*, *Clostridia*, *Nitrospirae*, *Thermodesulfobacteria*, *Thermodesulfobiaceae*) *and Archaea* (*Euryarchaeota* and *Crenarchaeota*) [52]. So far the known sulfate reducing archaea (SRA) include *Archaeoglobus* sp. (belonging to *Euryarchaeota*) from deep sea hydrothermal systems or oil reservoirs with optimal temperature up to 80 °C [3,10,53,54,55] and *Thermocladium modestius* and *Caldivirga maquilingensis* (belonging to *Crenarchaeota*) from acidic hot springs in Japan and the Philippines, respectively [56,57]. The absence of SRA-like *dsrB* gene sequences in this study could be ascribed to the different geochemistry (e.g., pH) between the studied hot springs and the geothermal features where the known SRA were retrieved.

The SRP populations in the studied Tibetan hot springs were diverse and mainly composed of *Deltaproteobacteria* (such as genera of *Desulfobacterales*, *Desulfovibrionales*, *Syntrophobacterales* and three unclassified *deltaproteobacterial* groups), *Clostridia* and *Nitrospirales*, with *Deltaproteobacteria* being most abundant. Such predominance of deltaproteobacterial SRB was consistent with previous studies in mesophilic temperature environments such as mangrove sediments [58,59,60,61], salt marsh [62], lake sediments [63,64] and high-temperature environments such as hot spring and hydrothermal systems [65,66]. However, at the order level the SRB population composition varies among different environments. For example, in Brazilian mangrove sediments, the deltaproteobacterial SRB population was mainly composed of *Desulfobacterales* and *Desulfovibrionales* [61]; in one estuarine salt marsh of China, the SRB population mainly consisted of *Desulfobacterales*, *Desulfovibrionales, Desulfarculales, Syntrophobacterales* and *Clostridialese* [62]; in Mono Lake sediments, SRB were dominated by *Deltaproteobacteria* with the order *Desulfovibrionales* being dominant [63,64]; in wetland sediments, SRB were dominated by *Desulfobacteraceae* with genera of *Desulfobacterium*, *Desulfobacca*, *Desulfococcus,* and a poorly-resolved group being dominant [67]. Thus, the observed differences in the composition of the SRB population between the studied Tibetan hot springs and the mesophilic environments mentioned above are quite natural. So far only one deltaproteobacterial *Thermodesulforhabdus norvegicus* strain A8444 has ever been isolated from hot environment (North Sea oil field water) and its growth temperature ranged 44–74 °C with an optimum at 60 °C [68]. However, little is known about characterized deltaproteobacterial SRP from hot springs. The present study expands our understanding on the distribution of sulfate reducing *Deltaproteobacteria* in hot springs.

It is notable that none of the obtained SRB clone sequences in this study showed a close relationship with the characterized SRB strains. At the species level, a lot of novel SRB strains have been isolated from terrestrial hot springs globally [15]. For example, *Thermodesulfovibrio yellowstonii* was isolated from thermal vent water in Yellowstone Lake, Wyoming, USA, and it grew at 40–70 °C with an optimum at 65 °C [69]; *Thermodesulfobacterium hveragerdense* and *Thermodesulfovibrio islandicus* strain R1Ha3 were isolated from microbial mats collected from an Icelandic hot spring and it grew at 55–74 °C with an optimum of 70–74 °C [70]; *Thermodesulfobium narugense* was isolated from a hot spring in Narugo, Japan [71]; *Thermodesulfobacterium commune* isolated from the Ink Pot spring in Yellowstone National Park, WY. grew at a temperature range of 45–80 °C with an optimum growth at 70 °C [72]. In addition, In the acid-sulfide hot spring of New Zealand, the observed sulfate reducers were affiliated with genera *Thermodesulfovibrio*, *Desulfovibrio*, and *Syntrophobacter* [65,66]. However, none of the obtained SRB clone sequences in the present study showed close relationship with the above mentioned SRB strains characterized so far. These results suggested that the Tibetan hot springs may be inhabited by unique SRB populations.

### 4.2. Temperature Response of dsrB Gene Diversity and Abundance in the Tibetan Hot Springs

Temperature has a fundamental impact on the metabolic rates of microorganisms and strongly influences microbial ecology and biogeochemical cycling in the environment [73]. Fishbain et al. deduced that the SRP community composition may be more closely linked to chemical parameters than to temperature, and thus SRP diversity is likely restricted at high temperature in hot springs [17]. Thermophiles are organisms that grow best at temperatures above 45 °C [73,74], and inhabit high temperature environments such as deep-sea vents and terrestrial hot springs. Microbial diversity is controlled by temperature in geothermal environments [75]. It is noteworthy that significant correlation was observed between the *dsrB* gene OTU numbers and temperature along a spring channel I (Figure 2B). In the present study, the OTU numbers of *dsrB* genes in a spring channel were significantly correlated with temperature. In the Arctic sediment, the SRB community was sensitive to temperature increases over 10 °C, and the steady decrease in microbial cells and the relative contribution of SRB to total microbial count with increasing incubation temperature implies that most of the SRB community was negatively affected by prolonged incubation temperatures of 10 °C and 20 °C [36]. Robador et al. reported that the structure of the SRM community is determined by temperature in polar, temperate, and tropical marine sediments [73]. On the other hand, the *dsrB* gene was most abundant at an environmental temperature of 56 °C. The minimum temperature (Tmin) and maximum temperature (Tmax) limit the growth range, and the optimum temperature (Topt) denotes the temperature at which the growth rate is maximum. We speculate that there is also “optimum temperature” for thermophilic SRB community in the studied Tibet geothermal zone, which awaits further investigation.

### 4.3. Sulfate Concentration Was Important Factor Shaping the Distribution of dsrB Gene Diversity in the Tibetan Hot Springs

Environmental factor can strongly affect the distribution and diversity of the microbial community [76]. The significantly different composition of SRP communities among the samples indicates that environmental conditions exert significant selection pressure on SRPs and lead to the formation of communities that are adapted to certain conditions [77]. Sulfate is the dominant sulfur species at 28 mM in the modern oxic oceans [9]. Marine sediments represent the greatest SRP richness (>25 *dsrB* families), suggesting that sulfate and DOC concentrations are the main determinant of distribution [78]. The concentration of sulfate in freshwater ranges from ∼10 to >500 μM, which is much lower than in seawater (28 mM) [79]. The concentration of sulfate in QZM hot springs reaches 5 mM and is a typical sulfate-rich environment for them. Many geochemical factors can affect the sulfate-reducing community in different environments. For example, salinity and ammonium ions are the environmental factors that best correlated with the sulfate reducer community from lagoons in the Amvrakikos Gulf (Ionian Sea, Western Greece) [80]; Ferric iron was the major factor controlling the structure of sulfate-reducing bacterial community across varied vegetations, and sulfate was closely correlated with the sulfate-reducing bacteria communities from *S. alterniflora* at both high and low tidal zones [62].

Sulfates are electron acceptors for sulfate-reducing bacteria [53]. Sulfate additionally supported some of the highest sulfate reduction rates ever measured in terrestrial aquatic environments [67]. SRB numbers were high primarily in the high-sulfate waters [81], increasing numbers of SRB in the water column were associated with higher sulfate input [82]. Sulfate concentration was considered an important environmental factor affecting the rate of sulfate reduction and the abundance of SRB. On the other hand, the sulfate concentration also affected the diversity of the sulfate-reducing community, with a low sulfate concentration, the *Firmicutes*-like group was the predominant sulfate reducer at 0.8 mbsf in the SMTZ [29]. Sulfate concentration was important factor shaping the distribution of *dsrB* gene diversity in the Tibetan hot springs.

## Figures and Tables

**Table 1:** Geographical and Geochemical Parameters of the Investigated Hot Springs in This Study.

Site	Characteristic	Sample	GPS Location (N/E)	Altitude (m)	pH	Temp. (°C)	Fe^2+^ (mg/L)	DO (ug/L)	DOC (mg/L)	TOC	SO_4_^2−^ (mg/L)
Daggyai (DG)	Separated	DG-1	85.7506°/29.5985°	5058	8.0	82.0	0.07	116	90.46	0.852%	83.4
		DG-4	85.7509°/29.5982°	5057	6.8	45.5	0.02	2100	60.68	0.555%	76.5
		DG-5	85.7509°/29.5982°	5057	7.5	32.2	0.03	3900	30.88	0.448%	47.9
		DG-14	85.7492°/29.6018°	5082	7.4	50.0	0.2	n.d	7.83	1.180%	8.4
		DG-16	85.7492°/29.6017°	5075	8.0	35.0	0.01	n.d	9.66	0.733%	62.4
Quzhuomu (QZM)	Channel I	QZM-4	91.8086°/28.2482°	4505	6.5	62.0	0.05	204	30.30	1.380%	327.8
		QZM-5	91.8086°/28.2482°	4505	6.8	56.0	0.34	1850	50.64	1.340%	492.3
		QZM-6	91.8086°/28.2482°	4505	7.0	54.0	0.29	2000	27.00	3.500%	460.6
		QZM-7	91.8086°/28.2482°	4505	6.8	52.0	0.15	3100	28.00	8.380%	431.9
	Channel II	QZM-9	91.8037°/28.2486°	4450	7.0	67.0	0.91	213	36.72	1.300%	461.2
		QZM-10	91.8037°/28.2486°	4450	7.0	65.0	0.57	496	33.61	1.980%	252.7
		QZM-11	91.8037°/28.2486°	4450	6.8	64.0	0.57	617	20.60	2.540%	417.9
		QZM-12	91.8037°/28.2486°	4450	6.8	62.0	0.52	1350	48.78	1.220%	381.5
	Separated	QZM-13	91.8034°/28.2485°	4438	6.7	65.7	0.33	561	958.60	1.020%	350.2
		QZM-14	91.8097°/28.2472°	4502	6.5	67.5	2.39	645	111.60	0.864%	354.7

n.d.: not detected.

**Table 2:** Ecological estimates of the *dsrB* gene libraries of the investigated hot spring sediments in this study.

Sample	No. of Clones	No. of OTUs	Coverage	Simpson (1/D)	Shannon (H)
QZM-4	25	2	100.0%	0.50	0.69
QZM-5	31	7	90.3%	0.75	1.60
QZM-6	35	9	91.4%	0.83	1.94
QZM-7	42	12	90.5%	0.86	2.17
QZM-9	65	12	92.3%	0.82	2.03
QZM-10	24	5	91.7%	0.63	1.20
QZM-11	25	4	96.0%	0.67	1.19
QZM-12	33	8	90.9%	0.82	1.84
QZM-13	19	4	100.0%	0.60	1.14
QZM-14	35	8	91.4%	0.69	1.55
DG-1	58	4	96.6%	0.38	0.68
DG-4	23	2	100.0%	0.16	0.30
DG-5	24	2	100.0%	0.49	0.68
DG-14	37	6	94.6%	0.72	1.42
DG-16	21	7	90.5%	0.75	1.66

## Data Availability

The data presented in this study are available on request from the corresponding author.

## Supplementary Materials

The following are available online at https://www.mdpi.com/2076-2607/9/3/583/s1, Table S1: Geochemical parameters of the investigated hot springs in this study; Table S2: The *dsrB gene* clones from the investigated hot springs and their closest relatives in the GenBank; Figure S1: Neighbor-joining tree showing the phylogenetic relationships of the deduced *DsrB* protein sequences translated from *dsrB* gene clone sequences obtained in this study to closely related sequences from the GenBank database. One representative clone type within each OTU is shown, and the number of clones within each OTU is shown in parentheses. The sequences from this study are shown in bold type, and they are coded as follows for the example of QZM-4: *dsrB* amino acid sequences of clone No. 4 from the Quzhuomu hot spring (QZM) sediment in Tibetan. The scale bar indicates the Jukes-Cantor distance. Bootstrap values of (1000 replicates) >50% are shown.
