# Supplementary material for: Influence of Temperature and Sulfate Concentration on the Sulfate/Sulfite Reduction Prokaryotic Communities in the Tibetan Hot Springs
Source: Microorganisms. 2021 Mar 12;9(3):583. doi: 10.3390/microorganisms9030583 (PMC8002027; doi:10.3390/microorganisms9030583)

Table S1 Geochemical parameters of the investigated hot springs in this study

Sample	Cl^- (mg L^{-1})	NO_2^- (mg L^{-1})	SO_4^{2-} (mg L^{-1})	Br (mg L^{-1})	NO_3^- (mg L^{-1})	Li (mg L^{-1})	Na (mg L^{-1})	NH_4^+ (mg L^{-1})	K (mg L^{-1})	Mg (mg L^{-1})	Ca (mg L^{-1})
-1	156.8	50.2	83.4	15.9	32.8	9.2	658.7	16.7	55.9	11.3	51.3
DG-4	155.9	36.3	76.5	9.9	27.7	10.6	772.3	16.7	74.1	5.1	49.7
DG-5	167.9	60.5	47.9	9.6	27.5	12.6	1055.7	35.2	126.0	8.9	91.9
DG-14	156.2	29.8	8.4	n.a.	26.6	9.6	659.3	5.4	70.7	3.6	20.4
DG-16	160.1	38.1	62.4	9.5	27.2	11.8	817.7	16.6	90.0	3.5	21.9
QZM-4	277.7	24.3	327.8	n.d.	28.8	3.9	291.2	28.5	47.9	38.3	316.1
QZM-5	273.7	28.0	492.3	11.7	30.3	3.9	284.0	28.0	60.0	48.3	536.5
QZM-6	282.1	62.9	460.6	n.d.	28.6	3.9	297.2	43.4	63.5	47.7	431.8
QZM-7	285.9	54.6	431.9	12.1	29.9	3.7	292.4	58.7	65.9	38.1	142.9
QZM-9	212.5	23.8	461.2	n.d.	30.0	2.6	187.9	3.0	21.2	33.7	264.6
QZM-10	128.6	19.6	252.7	n.d.	31.1	1.7	128.3	1.3	17.5	18.4	138.3
QZM-11	214.2	28.2	417.9	n.d.	30.8	2.8	202.1	3.2	24.5	39.6	328.0
QZM-12	208.8	31.1	381.5	n.d.	30.7	2.8	210.3	6.6	30.5	30.9	252.8
QZM-13	105.9	41.1	350.2	n.d.	32.7	1.3	112.6	8.2	23.8	39.8	259.1
QZM-14	208.4	21.8	354.7	n.d.	32.4	2.4	162.0	0.8	19.8	26.6	188.9

Table S2 The *dsrB gene* clones from the investigated hot springs and their closest relatives in the GenBank

OTU ID	QZM -4	QZM -5	QZM -6	QZM -7	QZM-9	QZM-10	QZM-11	QZM-12	QZM-13	QZM-14	DG-1	DG-4	DG-5	DG-14	DG-16	GenBank accession No. of the closest reference	Identity (%)
**OTU1**	12	5	4	5	3	1	11	8	2	4	12	2	0	0	0	AWH63881.1	93%
**OTU2**	13	12	10	10	3	0	0	1	0	0	0	0	0	0	0	AGJ00858.1	95%
**OTU3**	0	4	7	6	2	0	0	0	0	0	0	0	0	0	0	AFK32735.1	95%
**OTU4**	0	7	4	8	7	0	0	0	0	0	0	0	0	0	0	AWH64713.1	93%
**OTU5**	0	1	0	0	0	0	0	0	0	0	0	0	0	0	0	APA21667.1	91%
**OTU6**	0	0	1	0	0	0	0	0	0	0	0	0	0	0	0	AHZ11304.1	94%
**OTU7**	0	0	1	2	0	0	0	0	0	0	0	0	0	0	0	AWH63868.1	87%
**OTU8**	0	0	4	0	0	0	0	0	0	0	0	0	0	0	0	WP_018086255.1	99%
**OTU9**	0	0	1	1	0	0	0	0	0	0	0	0	0	0	0	AGR84841.1	92%
**OTU10**	0	0	3	2	0	0	0	0	0	0	0	0	0	0	0	ACO35898.1	97%
**OTU11**	0	0	0	3	0	0	0	0	0	0	0	0	0	0	0	AWH64381.1	90%
**OTU12**	0	0	0	1	0	0	0	0	0	0	0	0	0	0	0	ABX65363.1	96%
**OTU13**	0	0	0	1	0	0	0	0	0	0	0	0	0	0	0	AHZ11364.1	97%
**OTU14**	0	0	0	0	14	3	6	5	3	4	0	0	0	0	0	AWW01246.1	93%
**OTU15**	0	0	0	0	1	0	0	0	0	0	0	0	0	0	0	AWH64232.1	94%
**OTU16**	0	0	0	0	20	13	7	4	11	18	0	0	0	0	0	AWH63823.1	93%
**OTU17**	0	0	0	0	1	0	0	0	0	0	0	0	0	0	0	ACQ73016.1	93%
**OTU18**	0	0	0	0	1	0	0	0	0	0	0	0	0	0	0	AWH64728.1	96%
**OTU19**	0	0	0	0	2	0	0	1	3	4	0	0	0	0	0	AAO61660.1	95%
**OTU20**	0	0	0	0	9	6	1	7	0	0	0	0	0	0	0	BAF34624.1	93%
**OTU21**	0	0	0	0	1	0	0	0	0	0	0	0	0	0	0	AWH63978.1	92%

Table S2 The *dsrB gene* clones from the investigated hot springs and their closest relatives in the GenBank (*Continued*)

OTU ID	QZM -4	QZM -5	QZM -6	QZM -7	QZM-9	QZM-10	QZM-11	QZM-12	QZM-13	QZM-14	DG-1	DG-4	DG-5	DG-14	DG-16	GenBank accession No. of the closest reference	Identity (%)
**OTU22**	0	0	0	0	0	1	0	6	0	2	0	0	0	0	0	BAF34624.1	90%
**OTU23**	0	0	0	0	0	0	0	1	0	0	0	0	0	0	0	AWH64728.1	94%
**OTU24**	0	0	0	0	0	0	0	0	0	1	0	0	0	0	0	KUK12289.1	94%
**OTU25**	0	1	0	2	0	0	0	0	0	0	0	0	0	0	0	AWH63881.1	94%
**OTU26**	0	0	0	1	0	0	0	0	0	0	0	0	0	0	0	AWH63938.1	95%
**OTU27**	0	0	0	0	0	0	0	0	0	1	0	0	0	0	0	WP_088553243.1	82%
**OTU28**	0	0	0	0	1	0	0	0	0	1	0	0	0	0	0	ACP30533.1	94%
**OTU29**	0	1	0	0	0	0	0	0	0	0	0	0	0	0	0	APA21669.1	94%
**OTU30**	0	0	0	0	0	0	0	0	0	0	44	21	10	8	2	AFK32735.1	95%
**OTU31**	0	0	0	0	0	0	0	0	0	0	1	0	0	0	0	AWH64662.1	94%
**OTU32**	0	0	0	0	0	0	0	0	0	0	0	0	14	0	0	AHZ11384.1	99%
**OTU33**	0	0	0	0	0	0	0	0	0	0	0	0	0	13	3	AFK32735.1	98%
**OTU34**	0	0	0	0	0	0	0	0	0	0	0	0	0	12	3	ABK90639.1	98%
**OTU35**	0	0	0	0	0	0	0	0	0	0	0	0	0	1	0	AWW01163.1	88%
**OTU36**	0	0	0	0	0	0	0	0	0	0	0	0	0	2	9	AFK32737.1	94%
**OTU37**	0	0	0	0	0	0	0	0	0	0	0	0	0	1	0	ALN40079.1	90%
**OTU38**	0	0	0	0	0	0	0	0	0	0	0	0	0	0	1	AGR84883.1	90%
**OTU39**	0	0	0	0	0	0	0	0	0	0	0	0	0	0	2	AWH64526.1	93%
**OTU40**	0	0	0	0	0	0	0	0	0	0	0	0	0	0	1	RPI94506.1	97%
**OTU41**	0	0	0	0	0	0	0	0	0	0	1	0	0	0	0	AFK32730.1	85%

Figure S1. Neighbor-joining tree showing the phylogenetic relationships of the deduced *DsrB* protein sequences translated from *dsrB* gene clone sequences obtained in this study to closely related sequences from the GenBank database. One representative clone type within each OTU is shown, and the number of clones within each OTU is shown in parentheses. The sequences from this study are shown in bold type, and they are coded as follows for the example of QZM-4: *dsrB* amino acid sequences of clone No. 4 from the Quzhuomu hot spring (QZM) sediment in Tibetan. The scale bar indicates the Jukes-Cantor distance. Bootstrap values of (1000 replicates) > 50% are shown.

Fig S1A

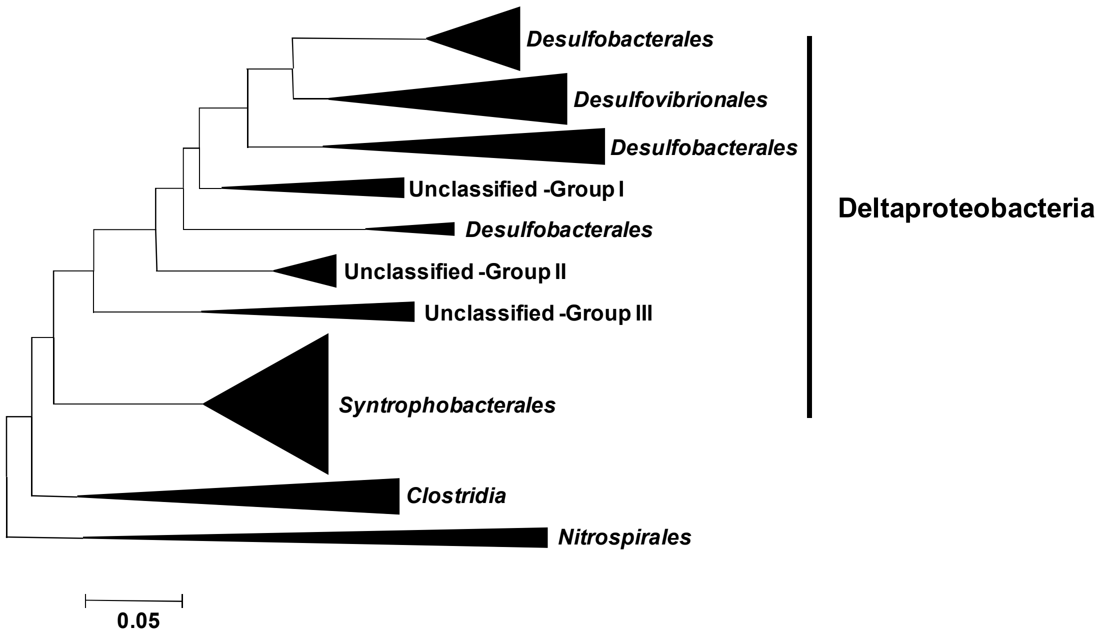

Fig S1B

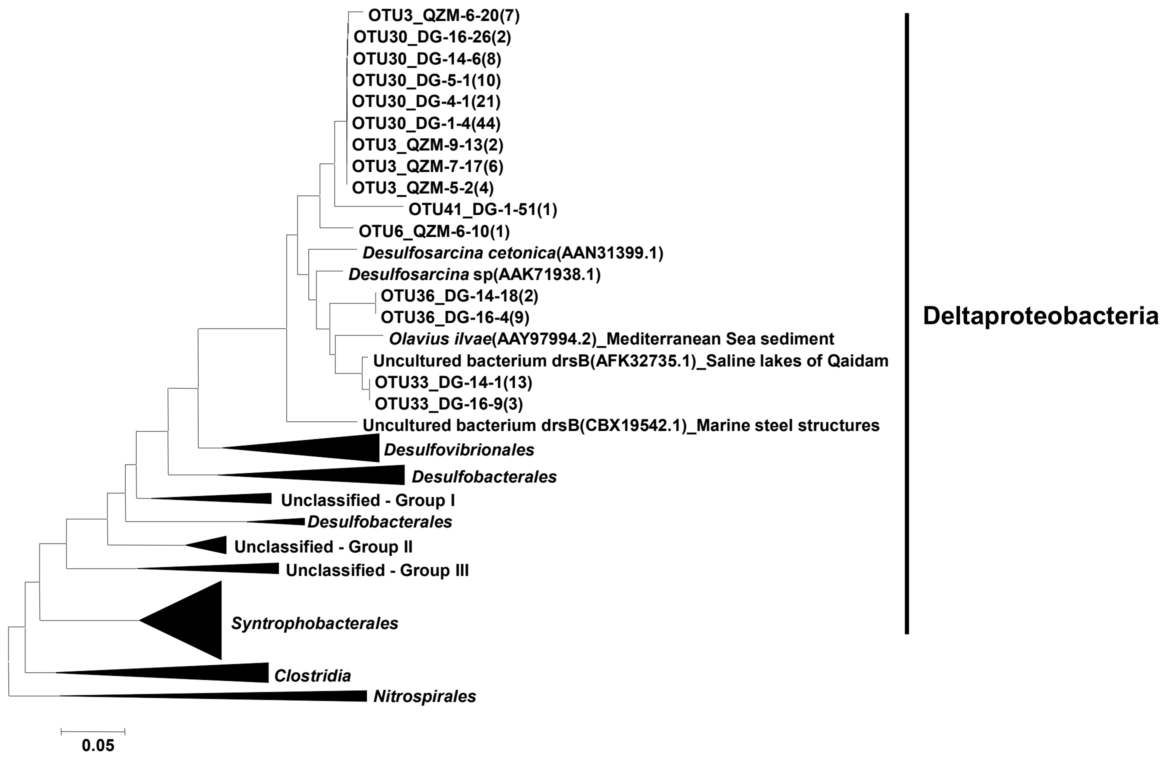

Fig S1C

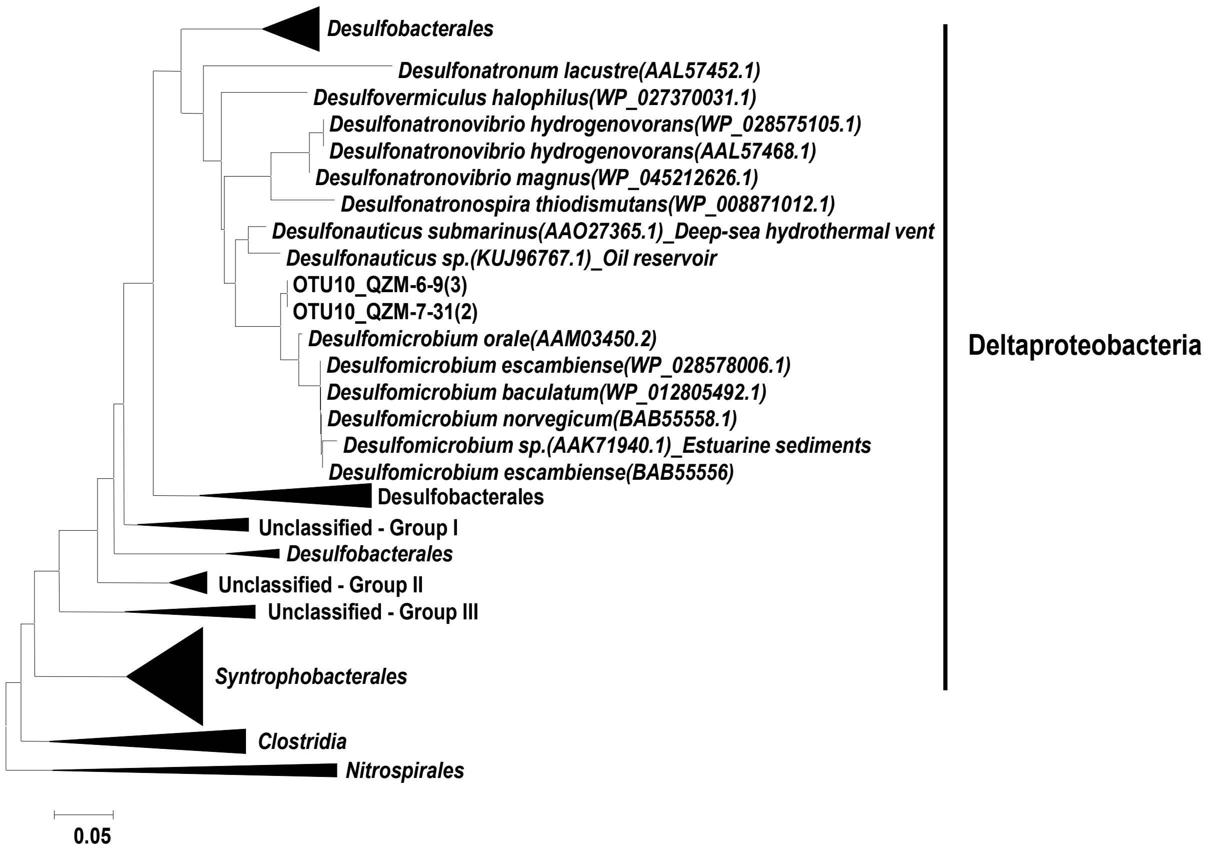

Fig S1D

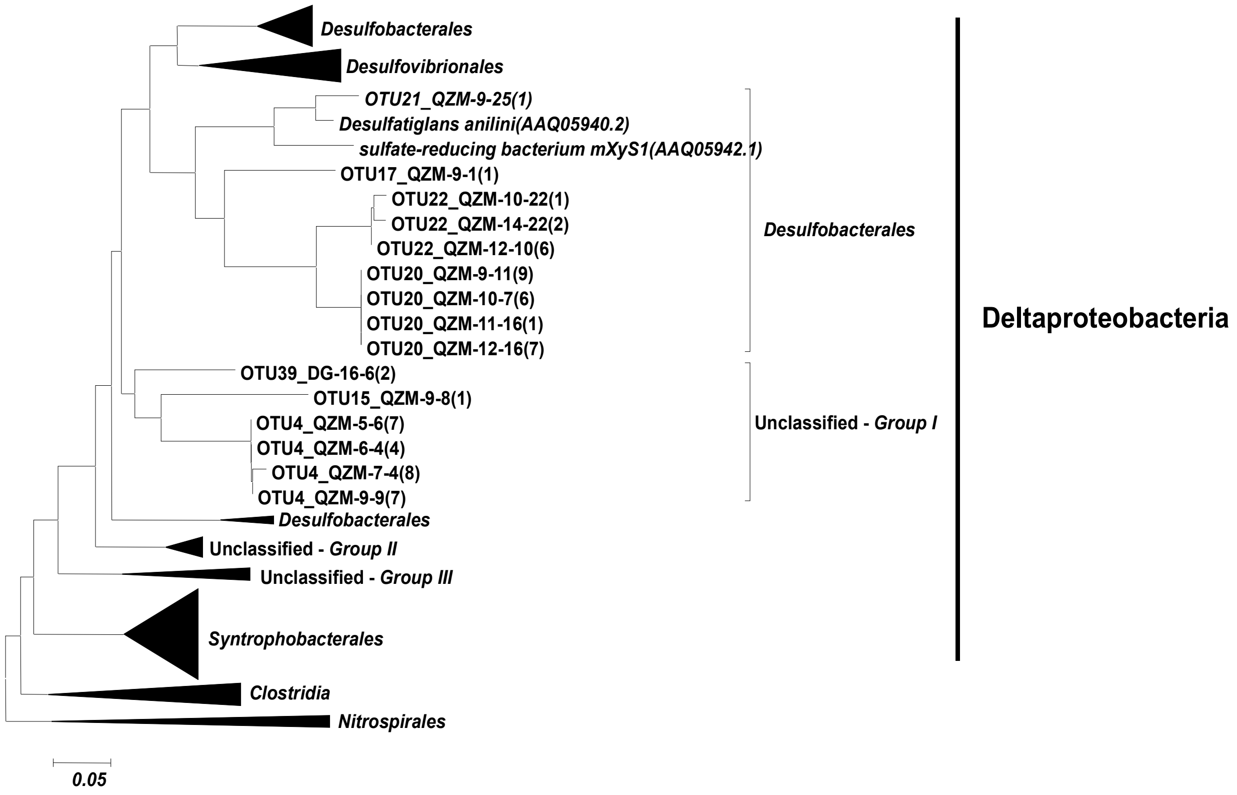

Fig S1E

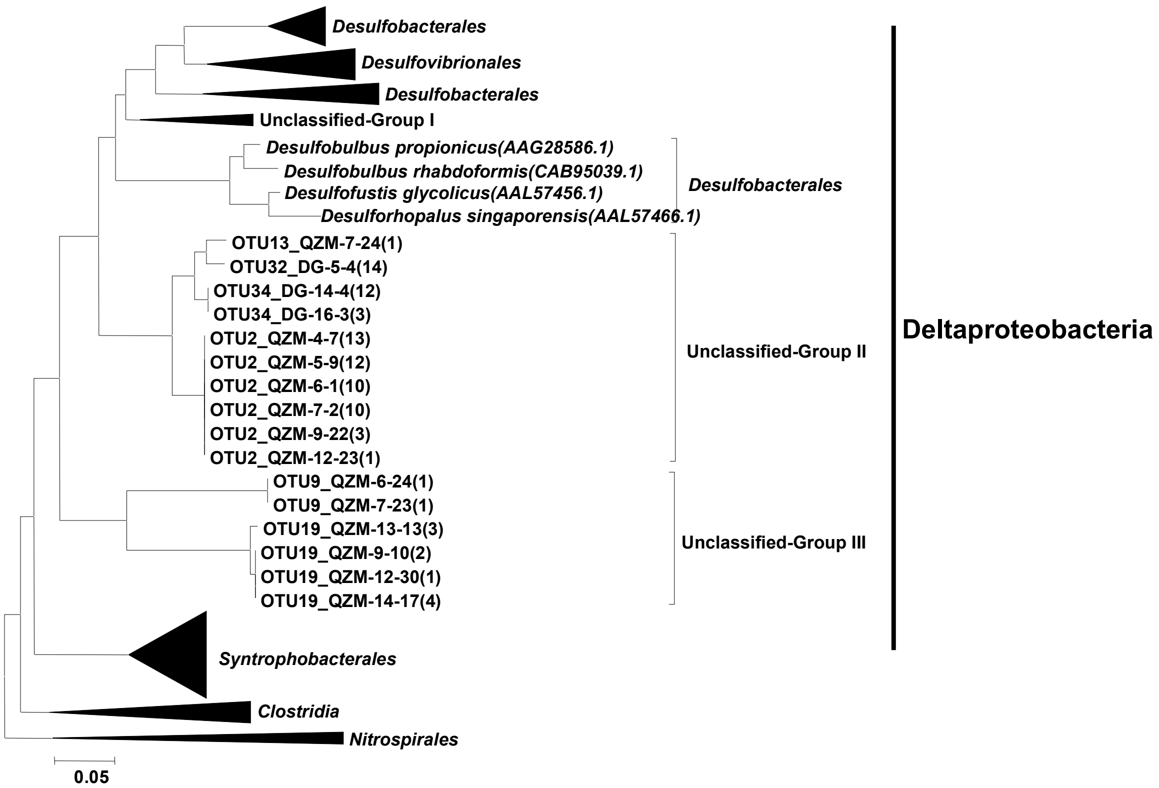

Fig S1F

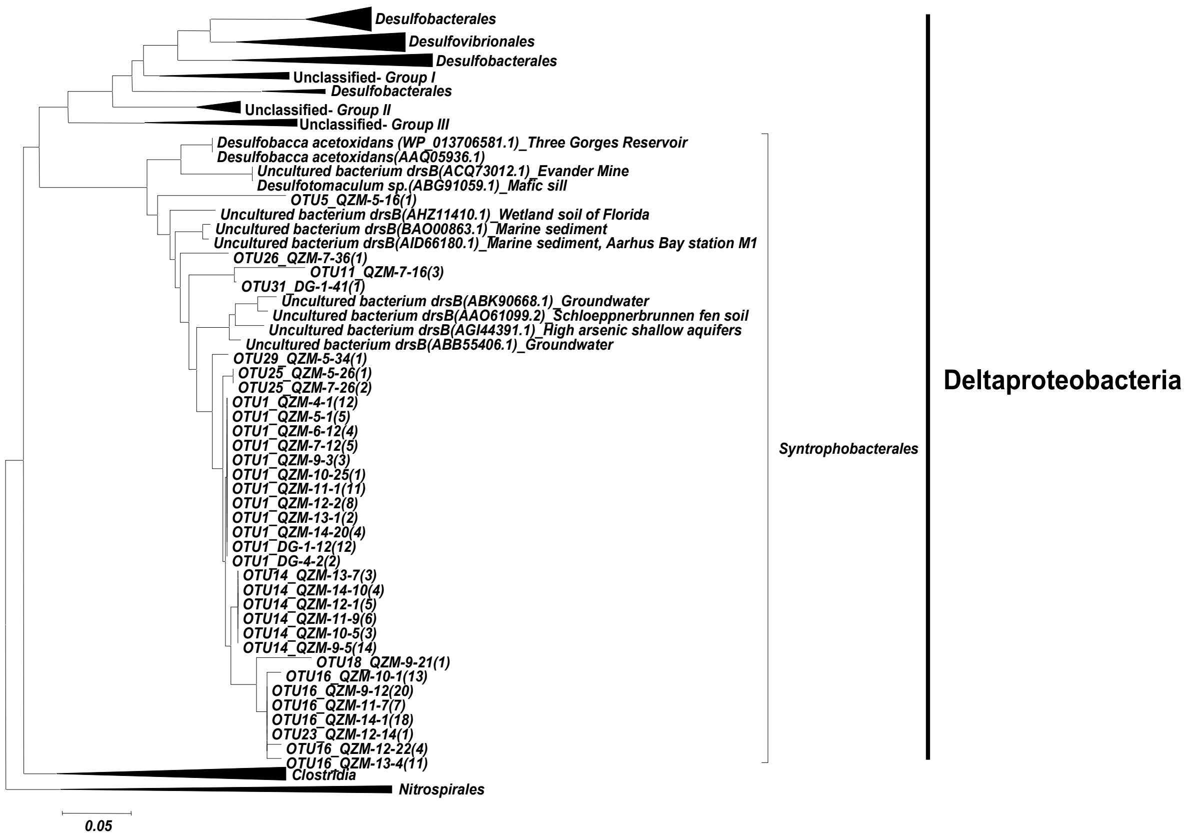

Fig S1G

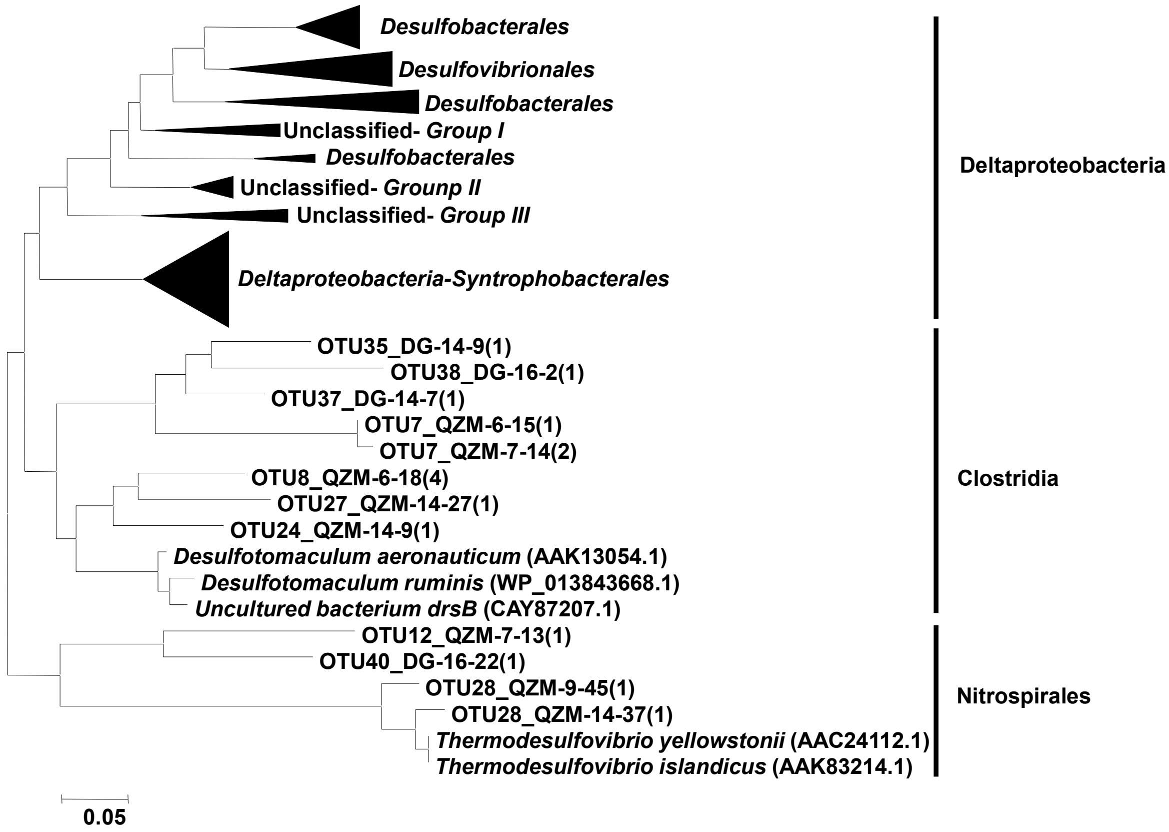

Supplement: Supplementary file 1 [file microorganisms-09-00583-s001.pdf]
